# Editorial Supplement—A Roaring Farce

**Published:** 1887-09

**Authors:** 


### [EDITORIAL SUPPLEMENT,]

### A ROARING FARCE.

[WITH A SERIOUS SIDE TO IT.]

*Montes parturiunt nascitur ridiculus mus !*

Since our article on the Asylum trouble was printed, and just as we go to press, (Saturday, Sept. 17th), we learn that the Board of Managers of the State Lunatic Asylum, after having rendered their verdict that the Superintendent was guilty of eleven of the charges against him,—and after having formally and officially notified him to that effect, and invited his resignation,—which request was participated in by all but one member, have since the Superintendent *refused* to *resign*, administered a mild reprimand, and, in effect, told him to go back to the Asylum and behave himself !—thus apparently, condoning the offenses of which (eleven of the graver charges), a majority of the board had convicted him ! We are reminded of the man who told another to " go to h—ll," and who, when called on for an apology. said : " Well, you need'nt go ;" or, of the little boy, who boasted to a companion, that he could make Dash, his dog, do whatever he commanded ; and when challenged for an illustration, stamped his foot, and said, "go out Dash !"—— "or under the bed," he added, as he saw Dash give unmistakable evidence of a determination to do the latter.

Here's a pretty how-de-do ! An appointee of the Board, who, the law says, may be removed for a good and sufficient cause, refuses to resign, upon request of the Board, coupled with notice of his conviction of conduct, unbecoming an officer of a public Institution, and though admitted to be "good and suffiicient cause," — the whole asylum being now, and for the last few weeks, or months, proven to be in a state of demoralization in consequence of it,—the Board, seemingly, has not the nerve or the power to enforce the law ; and thus the subordinate, defiant, stands triumphant, and the welfare of that great charity, made, seemingly, a secondary consideration of the will of the Superintendent, who, it will be remembered, was quoted on the trial as boasting that he was "solid with the Governor." The JOURNAL is loth to believe that Govervor Ross is the man to stand by an appointee, right or wrong, or to condone a course of conduct, which has notoriously scandalized the State, and destroyed at least for the time being, the efficiency of the service in one of the public Institutions,—yet how account for this strange action of the Board? It is said that lawyers have given an opinion that an unanimous vote of the Board is necessary to remove the Superintendent, even for cause; and that Dr. Dorset threatened, if removed by three, or even four of the members, to sue them for damages. It is also currently reported, and asserted in the secular press that the members backed down from their determination to declare the office vacant, "rather than incur litigation in a matter in which they had no personal interest." If this is true, the law is a farce, and under its construction one man may defeat, and in this instance seemingly has defeated, both its ends and objects, and the will of the majority; meantime, the *status quo* must obtain at the Asylum, and the Board remain mere figure heads—with no power to enforce their mandates; and the Governor it seems, (under the law), is equally impotent.

Had the Board pronounced the charges "not proven," the

JOURNAL would most sincerely have congratulated Dr. Dorset; but, so long as these grave allegations stand on the books, as *proven*, to the satisfaction of the Board, and their verdict of guilty so rendered, the JOURNAL fails to see either the sense or the justice of such a compromise; it is prejudicial to the welfare of the Institution, and a stigma on the 'scutcheon of the great State of Texas, as well as anything but creditable to the gentlemen who compose the Board. They thereby put themselves in a position of responsibility, seemingly shorn of corresponding power, and their will is apparently subordinated—notwithstanding Gov. Ross is quoted to have said that he would not interfere, but would abide the action of the Board. Gentlemen of the Board, your resignations are now in order; we do not believe this community, nor the people of Texas will endorse your action, under the circumstances.

We recall an incident of the trial, which is significant, and is illustrative of the old saying that "advice is easier to give, than to take;" Dr. Dorset is reported to have said to one of his employees: "Smith, I have tried every way to get you to do something for which you would be discharged,—why don't you resign like a gentleman, when you see I don't want you?"—Echo answers———

